# Relationship between β-Thalassemia minor and *Helicobacter pylori* infection

**DOI:** 10.22088/cjim.9.1.54

**Published:** 2018

**Authors:** Mohammad Zamani, Amin Vahedi, Ahmad Tamaddoni, Ali Bijani, Mojgan Bagherzade, Javad Shokri-Shirvani

**Affiliations:** 1 Student Research Committee, School of Medicine, Babol University of Medical Sciences, Babol, Iran; 2 Cancer Research Center, Health Research Institute, Babol University of Medical Sciences, Babol, Iran; 3 Non-Communicable Pediatric Diseases Research Center, Babol University of Medical Sciences, Babol, Iran; 4 Social Determinants of Health Research Center, Health Research Institute, Babol University of Medical Sciences, Babol, Iran; 5 Department of Immunology, Babol University of Medical Sciences, Babol, Iran; 6 Department of Internal Medicine, Rohani Hospital, Babol University of Medical Sciences, Babol, Iran

**Keywords:** *Helicobacter pylori*, thalassemia minor, prevalence

## Abstract

**Background:**

Until now, no study has been reported investigating the association between β-thalassemia minor and *Helicobacter pylori* (*H. pylori)* infection. This study was designed to compare *H. pylori* infection rate between β-thalassemia minor patients and healthy controls.

**Methods:**

A number of 100 β-thalassemia minor patients (50 males, 50 females) and 100 gender-matched healthy controls were prospectively recruited in this study in a period of 3 months. The study population consisted of the people who referred to a health center in Babol, North of Iran, for premarital counseling. *H. pylori* status was assessed by measuring the anti-*H. pylori* IgG antibodies using enzyme-linked immunosorbent assay. Demographic information and informed consent were collected from all participants.

**Results:**

The overall *H. pylori* infection rate was 43%. The infection was significantly more prevalent in thalassemia patients (53%) than in the controls (33%) in both univariate (OR=2.29, 95% CI: 1.3-4.06) and multivariable analyses (OR=2.05, 95% CI: 1.12-3.76). Age was the only significant factor which was positively correlated with the infection in β-thalassemia minor cases (OR=1.11, 95% CI: 1.02-1.2). Gender, blood groups, residency, and education level were not related to the infection.

**Conclusions:**

According to the results, it can be concluded that β-thalassemia minor patients are possibly more susceptible to *H. pylori* infection than healthy people. Further studies are needed to discover more about the exact mechanisms of increased susceptibility to *H. pylori* infection in β-thalassemia minor patients.

Thalassemia is a common genetic disorder characterized by a point mutation on globin gene expression(1). β-thalassemia is the result of impaired production of β-globin chains. This hemoglobinopathy has three principal forms, including thalassemia major, thalassemia intermedia and thalassemia minor (2). The clinical manifestations, such as mild microcytic and hypochromic anemia, are fully related to level of production of β-globin chain (3). The prevalence of β-thalassemia is seen more frequently in the Mediterranean countries, the Middle East and Central Asia (1, 4). *Helicobacter pylori (H. pylori)*, a gram-negative bacterium, colonizes the human gastric mucosa. This pathogen is the main cause of chronic gastritis, peptic ulcer, gastric cancer and dyspepsia (5, 6). The prevalence of *H. pylori* infection is seen in about half of the worldwide population and it is higher in the developing countries than in the developed countries (7, 8). Iran, a country, in the Middle East is an endemic region of thalassemia. About 20,000 homozygote β-thalassemia and 3,750,000 carriers have been identified in this country (9). All couples should be checked for β-thalassemia before marriage in Iran and those who are both carriers receive premarital counselling (10, 11). 

In North of Iran, β-thalassemia minor is the most frequent state of β-thalassemia and is estimated about 10% of the population(12). Taking into account the remarkable concurrent prevalence of β-thalassemia minor and *H. pylori* infection in northern Iran(10, 12, 13), and considering the high rate of gastric diseases among these thalassemia patients in this area, it appears that there may be an association between *H. pylori* and β-thalassemia minor. There have been some articles showing a probable relationship between *H. pylori* and recurrent abdominal pain (RAP) in people with hemoglobinopathies, such as sickle cell anemia and β-thalassemia (14, 15). 

In a survey by Karimi et al.(16), *H. pylori* infection in the β-thalassemia major patients with RAP was more common than in non-thalassemic controls with RAP. Although the difference was not significant, the authors suggested that *H. pylori* can possibly increase the risk of RAP in thalassemia patients (16). 

In the present study, we aimed to compare the prevalence of *H. pylori* infection between β-thalassemia minor patients and normal controls in Babol, north of Iran.

## Methods

Study population and data collection: This prospective study was performed on the people who refered to one of the health centers in Amirkola, Babol. This place is the major referral center for premarital thalassemia sreening in Babol.

A series of 100 persons who were confirmed diagnosis of β-thalassemia minor were consecutively included in the study (50 males, 50 females) during December 2015-February 2016. To confirm β-thalassemia minor, all participants' blood samples were checked initially for complete blood count (mean corpuscular volume<80 and mean corpuscular hemoglobin<27) and then for hemoglobin A2 using high-performance liquid chromatography (hemoglobin A2>3.5). 

An equal number of controls, whose genders were compatible, were enrolled in this survey. The recruited controls were those who referred to the same health center. Demographic information (age, gender, blood group, residency, and education level) was obtained from all individuals. 

Serum samples of the groups were collected and transmited to the immunology laboratory of Babol University of Medical Sciences and stored frozen at -20°C. To determine the infection, anti-*H. pylori* IgG antibodies were measured using enzyme-linked immunosorbent assay (ELISA) kits (IBL, Hamburg, Germany). Samples were considered positive for *H. pylori* when the antibody levels were more than 12 U/ml.

The study was approved by the Ethics Committee of Babol University of Medical Sciences and Health Services, the reference number is MUBABOL.REC.1395.71. Written informed consent was taken from all participants.

Chi-square and student's t-test were used to compare the demographic factors between case and control groups. To evaluate the association between *H. pylori* infection and β-thalassemia minor, we used the multivariable logistic regression analysis adjusted for residency and education by calculating odds ratio (OR) and 95% confidence interval (CI). The univariate analysis was performed to determine the relationship between the demographic variables and *H. pylori* infection in the β-thalassemia minor patients. A p<0.05 was considered statistically significant.

## Results

The mean age of β-thalassemia minor and control subjects was 25.21±5.92 (range, 14-46) and 26.12±7.13 (range, 14-68) years, respectively. Fifty-one percent of the individuals had blood type O, and majority of them (97%) were RH positive. Forty- eight percent of β-thalassemia minor patients and 65% of controls were urban inhabitants, and the difference was significant (P=0.015). There were significant differences between the two groups in the educational status and thalassemia subjects have lower level of education than controls (p<0.001) (table 1).

Overall, the prevalence of *H. pylori* was 43% in the study population. This rate was higher in β-thalassemia minor group (53%) compared with controls (33%), and the difference was significant (odds ratio [OR]=2.29, 95% CI: 1.3-4.06, P=0.005). This significant difference was observed between the groups even after adjustment for residency and education (OR=2.05, 95% CI: 1.12-3.76, P=0.02) (table 2).

Table 3 shows the association between *H. pylori* infection and risk factors in the β-thalassemia minor cases. Age was the only factor significantly positively associated with the infection (OR=1.11, 95% CI: 1.02-1.2). No significant correlation was found between *H. pylori* infection and other variables, including gender, ABO and Rh blood groups, residency, and education.

**Table 1:** Demographic characteristics of the participants

	**Thalassemia** **n(%)**	**Control** **n(%)**	**P-value**
**Gender**			1
Male	50 (50)	50 (50)	
Female	50 (50)	50 (50)	
Age (year)	25.21±5.92	26.12±7.13	0.327
**ABO Blood group**			0.972
A	15 (15)	17 (17)	
B	28 (28)	27 (27)	
AB	6 (6)	5 (5)	
O	51 (51)	51 (51)	
**Rhesus (Rh) blood** groups			1
Rh Positive	97 (97)	97 (97)	
Rh Negative	3 (3)	3 (3)	
**Residency**			0.015
Urban	48 (48)	65 (65)	
Rural	52 (52)	35 (35)	
**Education level**			0.0001
Below diploma	36 (36)	12 (12)	
Diploma	20 (20)	28 (28)	
Bachelor’s degree	44 (44)	60 (60)	

* 100 subject in each group

**Table 2:** Comparison of H. pylori infction between β-thalassemia minor and normal subjects

	***H. pylori*** ** positive** **N (%)**	**Univariate** **OR (95% CI)**	**P-value**	**Adjusted** a **OR (95% CI)**	**P-value**
Normal	33 (33)	1		1	
β-thalassemia minor	53 (53)	2.29 (1.3-4.06)	0.005	2.05 (1.12-3.76)	0.02

a Adjusted for residency and education. CI: confidence interval; OR: odds ratio

**Table 3:** Relationship between H. pylori infection and sociodemographic factors of β-thalassemia minor patients

	***H. pylori*** ** positive** **n(%)**	***H. pylori*** ** negative** **n(%)**	**OR (95% CI)**	**P-value**
**Gender **				
Male	30 (60)	20 (40)	1	
Female	23 (46)	27 (54)	0.57 (0.25-1.25)	0.16
Age	-	-	1.11 (1.02-1.2)	0.009
**ABO Blood group**				
A	9 (60)	6 (40)	1	
B	14 (50)	14 (50)	0.66 (0.18-2.37)	0.532
AB	4 (66.7)	2 (33.3)	1.33 (0.18-9.72)	0.777
O	26 (51)	25 (49)	0.7 (0.21-2.23)	0.539
**Residency **				
Urban	23 (48)	25 (52)	1	
Rural	30 (57.7)	22 (42.3)	1.49 (0.67-3.26)	0.329
**Education level**				
Below diploma	23 (64)	13 (36)	1	
Diploma	11 (55)	9 (45)	0.7 (0.22-2.1)	0.515
Bachclors degree	19 (43)	25 (57)	0.43 (0.17-1.06)	0.067

CI: Confidence Interval; OR: Odds Ratio

## Discussion

According to the results of this study, the prevalence of *H. pylori* infection was significantly higher in β-thalassemia minor patients than in the normal subjects. This is the first study reporting a significant association between β-thalassemia minor and *H. pylori* infection. In the past, few studies were performed to investigate the relationship between *H. pylori* infection and hemoglobinopathies, such as sickle cell anemia and β-thalassemia major, mostly based on determining the role of this pathogen in the occurrence of RAP in the patients with such diseases. Regarding sickle cell anemia, studies demonstrated controversial results about the relationship between* H. pylori* infection and this anemia (15, 17, 18). On the other hand, in relation to β-thalassemia major, surveys revealed no significant association between the infection and thalassemia. In their study, Karimi et al.(16) reported that the rate of *H. pylori* positivity in the β-thalassemia major patients with RAP was a little higher than in the healthy controls with the same symptoms (68% compared with 60%, p>0.05). Although the difference was not statistically significant, the authors proposed this pathogen as a potential cause of the RAP in the thalassemia patients(16). Also, the study by Balci et al.(14) indicated no significant differences between β-thalassemia major patients and non-thalassemic controls, both presenting with RAP, neither in serum anti-*H. pylori* IgG ELISA test (58.1% in comparison with 48.8%) nor in urea-breath test (48.4% compared with 39%). Nevertheless, there was not any report about the association of β-thalassemia minor with *H. pylori* infection and additional investigations are needed.

We believe that higher *H. pylori* infection rate in the β-thalassemia minor patients compared with controls in our study, may be related to the overload of iron. It is well-documented that iron is one of the essential factors for the pathogens to thrive in the host environment (19, 20), and *H. pylori* is no exception. Some published data proposed that *H. pylori* infection may cause iron-related diseases, such as anemia, iron deficiency, and iron deficiency anemia, in both children and adults (21-23), although other reports were not in accordance with these findings (24, 25). Considering that previous studies suggested a correlation between iron overload and increased risk of infections in the β-thalassemia patients (4, 26, 27), and also given that the iron excess can be seen mildly in the β-thalassemia minor patients(28, 29), it can be concluded that the overload of iron may lead to increase susceptibility to* H. pylori* infection. However, there is still no evidence supporting the hypothesis that iron overload is responsible for* H. pylori *infection in the β-thalassemia minor patients. So, more surveys should be done to uncover this dilemma.

In our study, it was observed that the seroprevalence of *H. pylori* had a positive correlation with age. This finding is in agreement with the previous literature showing the effect of birth cohort on *H. pylori* infection (30-32). By contrast, the prevalence of *H. pylori* was not different between two genders. There are no compatible findings on this topic. Moreover, the question of who is more susceptible to infection has remained open, that is, it is not clear if the infection occurred more frequently in males or in females (33-36). Regarding blood groups, no significant relationships were found between *H. pylori* positivity and blood groups ABO and Rh in the present study. While some previous reports alluded to these correlations (37, 38), others refuted them (39, 40). 

Besides, there were no associations found between *H. pylori* infection and residency and education. With regard to this subject, previous surveys indicated that low socioeconomic status can increase the risk of infection. Yet, there were inconsistent results whether the infection rate is higher in inhabitants of rural areas or in people living in the cities (41, 42). Furthermore, *H. pylori* infection has been identified less frequently in people with higher levels of education than persons with lower education in most of the studies (43-45). These different findings show that high literacy of the individuals can cause them to pay more attention to health and hygiene issues. 

A limitation of our survey was that the sample size was relatively small for assessing the relation between infection and variables in the thalassemia group. Definitely, the number of sample size was enough for evaluating the main purpose of the study, given that a significant correlation was found between beta-thalassemia minor and *H. pylori* infection.

In conclusion the results indicated that β-thalassemia minor patients are about two times more at risk of *H. pylori* infection in comparison with controls. The infection was significantly associated with age and not with type of blood, residency and education among the thalassemia patients. Further studies are needed to be designed for a more precise investigation of the mechanisms leading to increased susceptibility to *H. pylori* infection in β-thalassemia minor patients.

## References

[1] Cao A, Galanello R (2010). Beta-thalassemia. Genet Med.

[2] Thein SL (2013). The molecular basis of β-thalassemia. Cold Spring Harb Perspect Med.

[3] Nienhuis AW, Nathan DG (2012). Pathophysiology and clinical manifestations of the β-thalassemias. Cold Spring Harb Perspect Med.

[4] Vento S, Cainelli F, Cesario F (2006). Infections and thalassaemia. Lancet Infect Dis.

[5] McColl KE (2010). Clininal practice. Helicobacter pylori infection. N Engl J Med.

[6] Mentis A, Lehours P, Mégraud F (2015). Epidemiology and diagnosis of Helicobacter pylori infection. Helicobacter.

[7] Eusebi LH, Zagari RM, Bazzoli F (2014). Epidemiology of helicobacter pylori infection. Helicobacter.

[8] Malfertheiner P, Megraud F, O'morain C (2016). Management of Helicobacter pylori infection-the Maastricht V/Florence consensus report. Gut.

[9] Hashemizadeh H, Noori R (2013). Premarital Screening of Beta Thalassemia Minor in north-east of Iran. Iran J Ped Hematol Oncol.

[10] Zeinalian M, Nobari RF, Moafi A, Salehi M, Hashemzadeh-Chaleshtori M (2013). Two decades of pre-marital screening for beta-thalassemia in central Iran. J Community Genet.

[11] Abolghasemi H, Amid A, Zeinali S (2007). Thalassemia in Iran: epidemiology, prevention, and management. J Pediatr Hematol Oncol.

[12] Miri M, Tabrizi Namini M (2013). Thalassemia in Iran in Last Twenty Years: the Carrier Rates and the Births Trend. Iran J Blood Cancer.

[13] Khedmat H, Karbasi-Afshar R, Agah S, Taheri S (2013). Helicobacter pylori Infection in the general population: A Middle Eastern perspective. Caspian J Intern Med.

[14] Balci YI, Aral YZ, Covut IE, Polat Y, Turk M, Acimis N (2011). The frequency of Helicobacter Pylori infection in Beta Thalassemia major Patients with recurrent abdominal pain. Pak J Med Sci.

[15] Woods KF, Onuoha A, Schade RR, Kutlar A (2000). Helicobacter pylori infection in sickle cell disease. J Natl Med Assoc.

[16] Karimi M, Imanieh MH, Ghiam AF, Hashemi Z (2005). Investigation of Helicobacter pylori infection in β-thalassaemia major patients with recurrent abdominal pain. Eur J Gastroenterol Hepatol.

[17] Kennedy L, Mahoney DH, Redel CA (1997). Helicobacter pylori gastritis in a child with sickle cell anemia and recurrent abdominal pain. J Pediatr Hematol Oncol.

[18] Senbanjo I, Akinbamig A, Diaku-Akinwumi I (2010). Helicobacter pylori infection among a pediatric population with sickle cell disease. J Natl Med Assoc.

[19] Ganz T, Nemeth E (2015). Iron homeostasis in host defence and inflammation. Nat Rev Immunol.

[20] Skaar EP (2010). The battle for iron between bacterial pathogens and their vertebrate hosts. PLoS Pathog.

[21] Queiroz DM, Harris PR, Sanderson IR (2013). Iron status and Helicobacter pylori infection in symptomatic children: an international multi-centered study. PLoS One.

[22] Zamani M, Masrour-Roudsari J, Zamani V (2017). Hematologic disorder: A manifestation of helicobacter pylori infection. Caspian J Intern Med.

[23] Soundaravally R, Pukazhvandthen P, Zachariah B, Hamide A (2013). Plasma ferritin and indices of oxidative stress in Helicobacter pylori infection among schoolchildren. J Pediatr Gastroenterol Nutr.

[24] Xie C, Xu LY, Li W (2014). Helicobacter pylori infection in Mongolian gerbils does not initiate hematological diseases. World J Gastroenterol.

[25] Noto JM, Gaddy JA, Lee JY (2013). Iron deficiency accelerates Helicobacter pylori-induced carcinogenesis in rodents and humans. J Clin Invest.

[26] Rahav G, Volach V, Shapiro M (2006). Severe infections in thalassaemic patients: prevalence and predisposing factors. Br J Haematol.

[27] Galanello R, Origa R (2010). Beta-thalassemia. Orphanet J Rare Dis.

[28] Mosca A, Paleari R, Ivaldi G, Galanello R, Giordano PC (2009). The role of haemoglobin A2 testing in the diagnosis of thalassaemias and related haemoglobinopathies. J Clin Pathol.

[29] Piperno A, Mariani R, Arosio C, Vergani A (2000). Haemochromatosis in patients with β‐thalassaemia trait. Br J Haematol.

[30] Okuda M, Osaki T, Lin Y (2015). Low prevalence and incidence of helicobacter pylori infection in children: a population‐based study in Japan. Helicobacter.

[31] Yucel O (2014). Prevention of Helicobacter pylori infection in childhood. World J Gastroenterol.

[32] Ozbey G, Dogan Y, Demiroren K (2013). Prevalence of Helicobacter pylori virulence genotypes among children in Eastern Turkey. World J Gastroenterol.

[33] Singh V, Trikha B, Nain CK, Singh K, Vaiphei K (2002). Epidemiology of Helicobacter pylori and peptic ulcer in India. J Gastroenterol Hepatol.

[34] Shi R, Xu S, Zhang H (2008). Prevalence and risk factors for Helicobacter pylori infection in Chinese populations. Helicobacter.

[35] Moayyedi P, Axon AT, Feltbower R (2002). Relation of adult lifestyle and socioeconomic factors to the prevalence of Helicobacter pylori infection. Int J Epidemiol.

[36] Agah S, Khedmat H, Ghamar-Chehred ME, Hadi R, Aghaei A (2016). Female gender and Helicobacter pylori infection, the most important predisposition factors in a cohort of gastric cancer: A longitudinal study. Caspian J Intern Med.

[37] Ansari SA, Khan A, Khan TA (2015). Correlation of ABH blood group antigens secretion with Helicobacter pylori infection in Pakistani patients. Trop Med Int Health.

[38] Rasmi Y, Makhdoomi K, Farshid S, Kheradmand F (2011). Seroprevalence of anti-helicobacter pylori and anticytotoxin-associated gene a antigen antibodies according to abo blood groups and rhesus status among hemodialysis patients. Iran J Kidney Dis.

[39] Aryana K, Keramati MR, Zakavi SR, Sadeghian MH, Akbari H (2013). Association of Helicobacter pylori infection with the Lewis and ABO blood groups in dyspeptic patients. Niger Med J.

[40] Petrović M, Artiko V, Novosel S (2010). Relationship between Helicobacter pylori infection estimated by 14C-urea breath test and gender, blood groups and Rhesus factor. Hell J Nucl Med.

[41] Ozaydin N, Turkyilmaz SA, Cali S (2013). Prevalence and risk factors of helicobacter pylori in Turkey: a nationally-representative, cross-sectional, screening with the 13 C-Urea breath test. BMC Public Health.

[42] Baingana RK, Enyaru JK, Davidsson L (2014). Helicobacter pylori infection in pregnant women in four districts of Uganda: role of geographic location, education and water sources. BMC Public Health.

[43] Cheng H, Hu F, Zhang L (2009). Prevalence of Helicobacter pylori infection and identification of risk factors in rural and urban Beijing, China. Helicobacter.

[44] Nouraie M, Latifi‐Navid S, Rezvan H (2009). Childhood hygienic practice and family education status determine the prevalence of Helicobacter pylori infection in Iran. Helicobacter.

[45] Tonkic A, Tonkic M, Lehours P, Mégraud F (2012). Epidemiology and diagnosis of Helicobacter pylori infection. Helicobacter.

